# Gray value measurement for the evaluation of local alveolar bone density around impacted maxillary canine teeth using cone beam computed tomography

**DOI:** 10.4317/medoral.24677

**Published:** 2021-06-20

**Authors:** Cansu Köseoğlu Seçgin, Hazal Karslıoğlu, Mehmet Özgür Özemre, Kaan Orhan

**Affiliations:** 1 DDS, Assist. Prof., Department of Dentomaxillofacial Radiology, Faculty of Dentistry, Baskent University, Ankara, Turkey; 2 DDS, Assist. Prof., Department of Dentomaxillofacial Radiology, Faculty of Dentistry, Mersin University, Mersin, Turkey; 3 DDS, PhD, Assist. Prof., Department of Dentomaxillofacial Radiology, Faculty of Dentistry, Baskent University, Ankara, Turkey

## Abstract

**Background:**

To investigate whether any relationship between local alveolar bone density and maxillary canine impaction using gray values from cone beam computed tomography.

**Material and Methods:**

The cone beam computed tomography images of 151 patients were retrospectively evaluated. Maxillary canine was defined as an impacted tooth when root formation was complete and the patient’s age older than 13 or the other side of the maxillary canine has completely erupted. Similarly, complete eruption was defined as the tooth in its expected occlusion and position. Using the cone beam computed tomography software, the region of interest which was 5 mm2 in area, was placed in the trabecular bone on cross sectional cone beam computed tomography images and the gray value measurements were recorded. After measuring the gray values of all the teeth, the images were grouped according to the field of view size. Comparison of the gray values of impacted and non-impacted teeth was made between images with the same field of view size.

**Results:**

A total of 151 patients, 101 (66.9%) female and 50 (33.1%) male, were included in the study. The mean age of the patients was 24.94 ±13.9. In images with a 40X40 field of view, the gray values of the impacted canine teeth were higher than the gray values of the non-impacted ones and statistically significant difference was found between them (*p*=0.003). However no statistically significant difference was found between the gray values of impacted and non-impacted canine teeth in 60x60 and 100x50 field of view (*p*=0.197, *p*=0.170, respectively).

**Conclusions:**

We suggest using the smallest field of view size when evaluating bone density using gray values from cone beam computed tomography images and we support the idea that the local increased bone density may influence on impaction.

** Key words:**Cone-beam computed tomography, tooth, impacted, bone density, maxilla, image processing, computer-assisted.

## Introduction

The maxillary canine is the second most commonly impacted tooth, after the third molar, with an incidence rate that ranges from 0.8% to 2.8% (1). Over the years, several local, systemic and genetic factors for canine impaction have been proposed; however, the exact etiology remains unknown (2). The genetic factors and absence of the lateral incisors are the two main explanations of impaction (2,3). Impaction is more common in female patients, and most of these impacted teeth are displaced palatally (4).

Two-dimensional images provide inadequate information for the evaluation of impacted teeth (5). Cone beam computed tomography (CBCT) is an accurate and reliable method that supplies three-dimensional images of dentomaxillofacial structures without superimposition (5). CBCT images have been used to find the exact location of the impacted teeth and radiographic predictors of canine impaction (4). The strongest predictors were canine angulation in reference to the lateral incisor, the cusp tip angulation in reference to the occlusal plane, and the overall crown position (2). However, limited studies have assessed the relationship between bone density (BD) and maxillary canine impaction. BD, is one of the most important characteristics of bone quality. Several imaging techniques have been used to assess BD in dentistry including two-dimensional radiographs such as panoramic and periapical, computed tomography (CT) and CBCT (6). Fractal analysis of bone tissue on panoramic or periapical radiographs, calibrated Hounsfield unit (HU) values acquired from CT and gray values (GVs) obtained from CBCT were possible measurement methods for evaluating BD (7).

The increasing clinical use of CBCT means that evaluating the BD using this technique is more important. GVs obtained from CBCT are used in an analog form as the HU values to determine BD (8). A thorough understanding of GVs is very important for dentists, especially dentomaxillofacial radiologists, orthodontists and oral surgeons. In the literature, GVs obtained from CBCT images were studied for BD assessments of dental implants, the diagnosis of dental ankylosis, and the diagnosis and differentiation of pathological lesions (7,9-11). Although many factors affect the GV, many studies found linear correlations between GV and HU and concluded that the GVs are useful for BD assessment (12-14).

Understanding the effect of the adjacent alveolar BD on the etiology of impacted canines may aid in diagnosis and treatment of the condition. In the literature, only one study focused on this topic, and it concluded that an increased BD may play a local etiologic role in maxillary canine impaction (4). Moreover, no study assessed the BD around the impacted maxillary canine teeth using GVs obtained from CBCT. The aim of this study was to investigate whether there was a relationship between the local alveolar BD and maxillary canine teeth impaction using the GVs from CBCT. The null hypothesis is that the local BD does not affect maxillary canine teeth impaction.

## Material and Methods

- Sample and assessment of CBCT images

The patient sample for this study was selected from the archives from the department of Dentomaxillofacial Radiology, Faculty of Dentistry, Baskent University. The CBCT images were acquired between 2017-2020 for various purposes unrelated to this study, such as evaluations of impacted teeth, orthodontic treatment and implant planning. From all 2160 CBCT scans taken between these years, only images showing impacted and/or non-impacted maxillary canine teeth were selected. A maxillary canine was defined as either an impacted tooth when the root formation is complete and the patient’s age older than 13 or when the other side of the maxillary canine has completely erupted (4). Similarly, complete eruption was defined as the tooth in its expected occlusion and position. The patients’ age and gender, the field of view (FOV) size of image, and position of the impacted canine teeth for the right and left sides were recorded. The inclusion criteria for the study were as follows: (1) a CBCT scan showing unilateral or bilateral maxillary canine impaction with a complementing clinical diagnosis, (2) the patient’s age being older than 13; and (3) no prior orthodontic treatment. The exclusion criteria were as follows: (1) presence of syndrome or systemic disease affecting bone health; (2) CBCT scans that displayed pathology; (3) congenitally missing teeth, supernumerary teeth, dentigerous cyst, or an enlarged cystic follicle; (4) history of dental trauma or anterior maxillary surgery; and (5) images of patients with motion or any significant artifact on the CBCT image. The CBCT images that met the inclusion criteria of this study were selected, images that met the exclusion criteria were excluded. Consequently, the final sample size was 151 CBCT images. This study was approved by Baskent University Institutional Review Board (Project no: D-KA 21/07).

**Figure 1:**
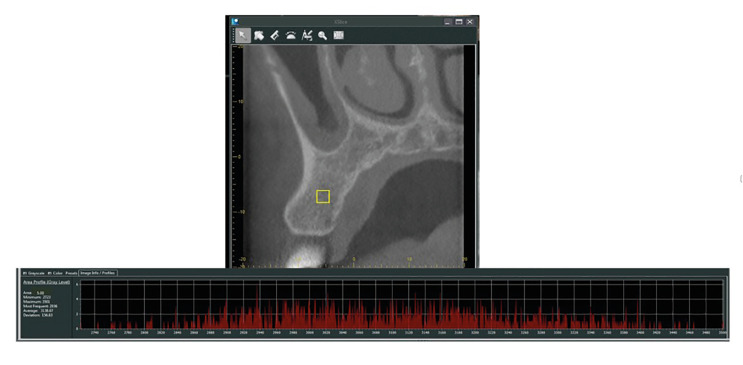
Selection of alveolar proces interproximal to the first and second premolars for measurement of GVs. ROI (5mm2) was placed in the trabecular bone on cross sectional CBCT images. The mean, minimum, and maximum GV measurements and standard deviations were automatically calculated by the software as shown.

All CBCT images were acquired by using Morita 3D Accuitomo 170 (J Morita, Kyoto, Japan) with the following parameters: 90 kVp, 5 mA, voxel size: 0.08, 0.125, 0.25 mm, FOV size: 40x40, 60x60 and 100x50 mm, respectively. The images were analyzed by using the i-Dixel software (v.2.2.1.6, Morita, Kyoto, Japan) on the medical monitor (Eizo Radiforce MX270W, Eizo Corporation, Ishika, Japan). Using the i-Dixel software, the region of interest (ROI) which had an area of 5 mm2, was placed in the trabecular bone on cross sectional CBCT images. ROIs were selected from areas that did not contain cortical bone or vascular canals. The maxillary alveolar process interproximal to the first and second premolars was selected as the ROI because of the availability of trabecular bone (4). The mean, minimum, and maximum GV measurements and standard deviations were automatically calculated by the software (Fig. 1). After measuring the GVs of all teeth that met the inclusion criteria in our study, the images were grouped according to the FOV size. A comparison of the GVs of both impacted and non-impacted teeth was made between images with the same FOV size. Because, GVs obtained from CBCT examinations were significantly affected by the FOV size. In small FOVs, the diameter of the x-ray beam becomes smaller to irradiate only the region of interest. With large FOVs, variability in GVs increases due to exomass, beam hardening artifact, and changes in patient position (15).

- Intra and interobserver reliability

All images were assessed by a dentomaxillofacial radiologist with 15 years experience. Fifty images were randomly selected from the sample and assessed twice for intraobserver agreement. There was an interval of one month between assessments. Regarding the interobserver agreement, the second observer was a dentomaxillofacial radiologist with five years’ experience who evaluated the same images. Both observers had previously been calibrated regarding the placement of the ROI. The intra- and interobserver agreements were assessed by intraclass correlation coefficient (ICC) analysis.

- Statistical analysis

A statistical analysis was conducted using SPSS (Version 22.0, SPSS Inc., Chicago, IL, USA) software. Descriptive statistics of the categorical variables were presented with numbers and percentages (%). Descriptive statistics of the continuous variables were presented with the median (min-max) and mean ± standard deviation (SD) depending on the data normality distribution. The normality distribution of the data was tested using the Shapiro-Wilk test. Since the parametric test assumptions were not provided, comparisons between two independent groups were performed using the Mann Whitney U test, and comparisons between two dependent groups were performed using the Wilcoxon test. The proportion comparisons and relationships between the categorical variables were conducted using the Chi-square test. The threshold level of statistical significance was *p*<0.05.

## Results

A total of 151 patients, of which 101 (66.9%) female and 50 (33.1%) were male, were included in the study. There were 71 impacted and 33 non-impacted canine teeth on the right side and there were 69 impacted and 27 non-impacted canine teeth on the left side. Of the 140 impacted teeth in total, 109 (77.9%) were mesioangular, 23 (16.4%) were vertical and eight (5.7%) were at the horizontal position. The mean age of the patients was 24.94 ±13.98 (min-max:14-83). The intra- and interobserver agreements were excellent (ICC ≥ 0.95 and 0.94, respectively).

**Figure 2:**
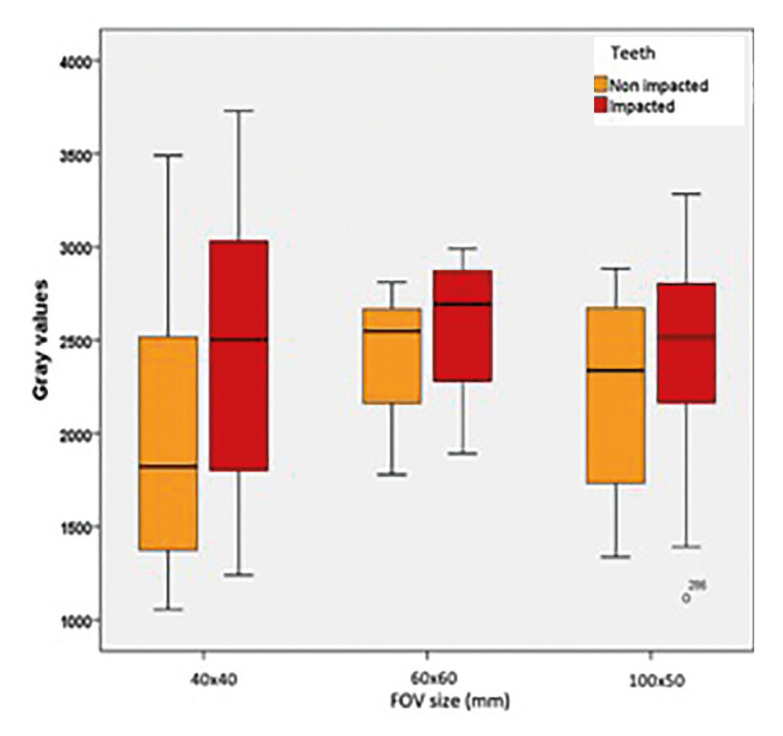
Boxplot of the distribution of GVs of impacted and not impacted canine teeth with different FOV sizes.

Descriptive statistics and statistical comparisons of the GVs of impacted and non-impacted canine teeth in images with the same FOV are presented in Table 1. In images with a 40X40 FOV, the GVs of the impacted canine teeth were higher than the GVs of the non-impacted ones and a statistically significant difference was found between them (*p*=0.003). However no statistically significant difference was found between the GVs of impacted and non-impacted canine teeth in 60x60 and 100x50 FOV (*p*=0.197, *p*=0.170, respectively). The distribution of the GVs of the impacted and non-impacted canine teeth in different FOV sizes is shown in Fig. 2.

**Figure 3:**
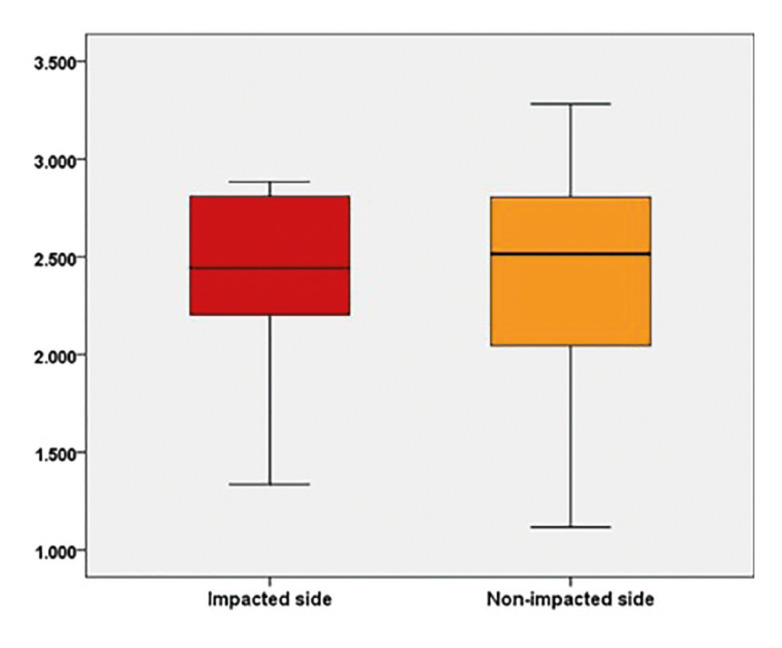
Boxplot of the distribution of GVs of the impacted and non-impacted sides in the same patient (n=18).

There were 18 patients with unilateral canine tooth impaction in images with 100x50 FOV (Table 2). No statistically significant difference was found between the GVs of impacted and non-impacted sides (*p*=0.703). The distribution of the GVs of impacted and non-impacted canine teeth in different FOV sizes is shown in Fig. 3.

**Table 1:**
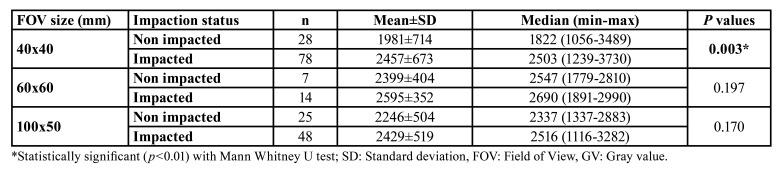
Comparison between GVs from CBCT images of impacted and non-impacted maxillary canine teeth with the same FOV size.

**Table 2:**
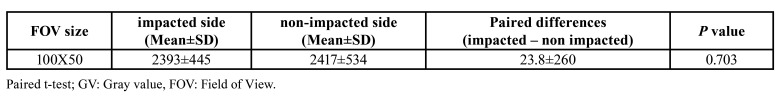
Comparison between the GVs of the impacted and non-impacted sides in patients with unilateral impacted canines (n=18).

The incidence rate of the impacted maxillary canine teeth was 62.1% in females and 37.9% in males. However, there was no statistically significant difference between females and males in terms of canine impaction (*p*=0.188).

## Discussion

Although researchers have focused on trying to identify the general and local etiological factors that are responsible for impacting maxillary canine teeth, the precise etiology is still unknown. However, previous studies have emphasized genetics and the absence of or anomalies in the lateral incisor as etiologic factors for maxillary canine impaction (3,16,17). The BD of maxillary canine is influenced by the extraction of adjacent teeth with or without socket preservation (18). In addition, to understand the effect of bone quality on impaction, the trabecular microarchitecture around impacted and non-impacted maxillary canine teeth has been assessed in one study, where they suggested that impaction may be affected by increased local BD (4). Nevertheless, the alveolar BD around the impacted maxillary canines has not been evaluated using GVs from CBCT as a potential etiologic factor for impaction.

In our study, effect of local alveolar BD on maxillary canine impaction was evaluated using GVs from CBCT. We found that the GVs of impacted teeth were significantly higher than the GVs of the non-impacted teeth in 40x40 mm FOV size (*p*=0.003). Servais *et al*. used CBCT to evaluate relationship of the unilateral and bilateral maxillary canine impactions and the microstructure of maxillary alveolar bone, as measured by the bone surface area and the bone fractal dimension (4). They found that the bone surface area was greater on the impacted side than the nonimpacted side. In addition, they demonstrated that bone marrow area had decreased more near the impacted canine compared to the non-impacted canine. Therefore, they concluded that increased BD may play a local etiologic role in maxillary canine impactions. Similarly, we found increased GVs around the impacted maxillary canines in the smallest FOV CBCT images.

Our findings showed that there was no statistically significant difference between the GVs of impacted and non-impacted canine teeth in 60x60 and 100x50 FOV (*p*=0.197, *p*=0.170, respectively). For images with 60x60 FOV, only 14 impacted and seven non-impacted teeth were analyzed. Due to the small sample size, a statistically significant difference may not have been found. In large FOVs such as 100x50 mm, the variability in GVs increases because of the exomass and changes to the positioning of the patient (15,19). Although the nature of our study was retrospective, all images were taken using the same device, and the comparisons of the GVs of impacted and non-impacted canine teeth were compared in images with the same FOV.

In the study sample, there were 18 patients with bilateral maxillary canines with unilateral impaction in images with 100x 50 FOV. No statistically significant difference was found between the impacted and non-impacted sides of the same patient in terms of the GV (*p*=0.703). On the contrary, Servais *et al*. found that the trabecular bone was denser at the sites of impaction (4). These results may be due to the different techniques used for the BD assessment and the small sample size.

The impaction of maxillary canine teeth is more common in female patients (20). However, there was no statistically significant difference between the females and males in terms of canine impaction in the present study (*p*=0.188).

In the literature, HU values obtained from CT and GVs obtained from CBCT images were used for BD assessments. In CT, the density of specific regions is determined by the HU (21). The HU represents the relative density of a body tissue according to a calibrated gray level scale based on HU values of the air (-1000 HU), water (0 HU) and dense bone (+1000 HU) (22). The term GV, indicates the level of brightness of a pixel (23). Considering the different natures of CT and CBCT, the HU of CT differs from the GV of CBCT. CT is rarely used in dentistry because CBCT has some advantages compared to CT, such as the lower equipment cost, lower patient radiation dose than CT, shorter scanning time, and a higher spatial resolution (22). Therefore, the analysis of BD using GVs is important because CBCT is commonly used for most dental procedures.

In dentomaxillofacial radiology, GVs obtained from CBCT can be used to determine BD for various reasons, such as BD around dental implants and bone graft evaluations (7). The correlation between GVs and HU values have been investigated by many researchers. Some researchers have reported incompatible results due to the large amounts of scattered X-rays and artifacts in CBCT scans (24). However, several studies have demonstrated a high correlation between the GV and HU, which suggests that CBCT can be used to estimate BD (6,25-27). In the present study, GVs obtained from CBCT images were used to assess alveolar BD around the maxillary canine teeth.

There are many recent studies found in the literature regarding assessing BD using CBCT to provide sTable reference values for jawbones (28-30). Andruch and Plachta investigated the clinical possibility of measuring the maxillary relative bone density through the clinical use of GVs using the CBCT radiological BD measurement tool. They found that there was a statistically significant difference between the radiological density of dental alveolus in the anterior and posterior maxilla (9). Hao *et al* assessed the bone density of dental implant sites using CBCT and established a quantitative ranges for each bone quality classification according to the classification of bone quality proposed by Lekholm and Zarb. They found that the anterior mandible had the highest mean BD, and posterior maxilla had the lowest BD (28). In the present study, the mean GV of the ROIs on CBCT was 1981±714 for the impacted teeth and 2457±673 for the non-impacted teeth in 40x40 FOV, respectively, and the difference between them was statistically significant (*p*=0.003). However, the measured GVs may be particular for each device because many factors may affect image quality and determination of GV in CBCT examinations, such as presence of artifacts, FOV size, ROI size, scanning parameters and variations in the devices (24). The GVs are highly influenced by device and scan settings (10). Therefore, assessing BD by measuring the GVs from CBCT should consider the scanning parameters, especially the FOV and ROI sizes.

To the best of our knowledge, this study is the first to assess BD around impacted maxillary canine teeth using GVs from CBCT. Images were obtained between the maxillary first and second premolars on the impacted and nonimpacted sides of the arch because the positions of the impacted canines are variable, and this area was the closest reproducible region for the measurements (4). There were potential limitations in the present study. First, the relatively small sample size due to the strict inclusion criteria of the study to create a homogenous sample. This is because our study sample consisted of no possible systemic or local known pathology affecting the maxillary canine teeth impaction, and all CBCT images in our sample had the same scan settings. Furthermore, the comparison of the GVs of impacted and non-impacted teeth was made using images with the same FOV size. Secondly, it should be elucidated that the GVs obtained from the used CBCT device could not be applied to other devices. Further studies with larger sample and using different CBCT devices are required to determine the role of the BD on maxillary canine impaction.

## Conclusions

In the present study, our analysis showed that the trabecular bone around impacted maxillary canines was denser than non-impacted ones in 40x40 mm FOV size. However, there was no statistically significant difference found between the GVs of impacted and non-impacted canine teeth in other FOV sizes (60x60 mm and 100x50 mm). According to the our results, we recommend using the smallest FOV size when evaluating BD using GV from CBCT images, and we support the idea that the local increased BD may have an influence on impaction. Further research using a larger sample size are required to determine the exact influence of BD on impaction.
